# Association of cigarette smoking with oral bacterial microbiota and cardiometabolic health in Chinese adults

**DOI:** 10.1186/s12866-023-03061-y

**Published:** 2023-11-17

**Authors:** Qiumin Huang, Xuemei Wu, Xiaofeng Zhou, Zhonghan Sun, Jie Shen, Mengmeng Kong, Nannan Chen, Jian-Ge Qiu, Bing-Hua Jiang, Changzheng Yuan, Yan Zheng

**Affiliations:** 1 School of Life Sciences and Human Phenome Institute, Fudan University, Shanghai, 200433 China; 2 School of Public Health, Fudan University, Shanghai, 200032 China; 3 School of Public Health, Zhejiang University School of Medicine, Hangzhou, 310058 China; 4 School of Medicine, Nantong University, Jiangsu, 226019 China; 5 The Academy of Medical Science, Zhengzhou University, Zhengzhou, 450000 China; 6 Zhongshan Hospital, Fudan University, Shanghai, 200032 China

**Keywords:** Oral microbiome, Cigarette, Smoking, Cardiometabolic health, Chinese adults

## Abstract

The interplay among cigarette smoking status, oral microbiota, and cardiometabolic health is poorly understood. We aimed to examine the association of cigarette smoking status with oral microbiota and to assess the association of the identified microbial features with cardiometabolic risk factors in a Chinese population. This study included 587 participants within the Central China Cohort, including 111 smokers and 476 non-smokers, and their oral microbiota was profiled by 16S rRNA sequencing. Both oral microbial alpha- and beta-diversity were distinct between smokers and non-smokers (*p* < 0.05). With adjustment for sociodemographics, alcohol and tea drinking, tooth brushing frequency, and body mass index, the relative abundance of nine genera and 26 pathways, including the genus *Megasphaera* and two pathways involved in inositol degradation which have potentially adverse effects on cardiometabolic health, was significantly different between two groups (FDR *q* < 0.20). Multiple microbial features related to cigarette smoking were found to partly mediate the associations of cigarette smoking with serum triglycerides and C-reactive protein levels (*p-*mediation < 0.05). In conclusion, cigarette smoking status may have impacts on the oral microbial features, which may partially mediate the associations of cigarette smoking and cardiometabolic health.

**Supplementary Information:**

The online version contains supplementary material available at 10.1186/s12866-023-03061-y.

## Introduction

The human oral cavity contains a diverse and dynamic community of microorganisms, including bacteria, archaea, fungi, viruses, and protozoa, collectively known as the oral microbiome [1]. It has been demonstrated that oral microbes participate in human digestion and protection against pathogen [2], and the dysbiosis of oral microbial communities has been linked to the development of periodontal and systemic diseases such as periodontitis and cardiovascular disease [3], [4].

The homeostatic balance of oral microbiome depends on various factors such as age, disease status, oral hygiene practices and lifestyles [5]. Cigarette smoking, potentially causing multiple system dysfunctions, is also a common determinant of the oral microbial ecology, due to interactions between smoking toxicants and oral microbes in the complex oral micro-environment [6]. Several epidemiological studies have focused on whether the oral microbiome structures varied by different smoking status, but findings were inconsistent [7–10]. For example, in a study of 1204 U.S. adults, current smokers showed a distinct microbial diversity compared to non-current smokers using oral wash samples [8], but such a difference was not observed in any of the eight oral sites investigated in a subsequent study including 23 current smokers and 20 never smokers [9]. Though multiple bacteria taxa were reported to be associated with cigarette smoking in these prior studies, the relationship of these identified oral microbial features with cardiometabolic health has not been further reported [7–10].

In China, public health burdens were greatly attributed to cigarette smoking consumption, especially with the increasing proportion of adult cardiovascular mortality resulting from smoking [11]. It is important to understand the potential interplays among cigarette smoking, oral microbiome, and cardiometabolic health, which may help reveal microbial mechanistic insight into the interventive role of cigarette smoking for cardiometabolic diseases. Therefore, in the present study, we aimed to examine the associations of cigarette smoking status with the oral microbial composition, individual taxon and pathway, by bacterial 16S ribosomal RNA (rRNA) gene sequencing. We further evaluated the potential associations of the identified microbial features with the circulating cardiometabolic risk factors.

## Results

### Characteristics of the study participants

The general profile of study participants’ characteristics is shown in Table 1. Among 587 participants included, 111 (18.9%) smoked regularly, with an average cigarette smoking pack-year of 23.03 (standard deviation 20.26). They tended to have higher average age, annual income, BMI, serum TG and CRP levels, lower education level and daily teeth brushing frequency, and were more likely to be alcohol and tea drinkers (all *p* < 0.05), compared to that of non-smokers. None significant difference in bedtime eating habits, chronic disease history, and antibiotic use was observed between two groups.

**Table 1:** Demographic characteristics of the study participants by smoking status

Characteristics	Non-smoker (N = 476)	Smoker (N = 111)	*p* value
Sex (Male)	85 (18%)	111 (100%)	< 0.001
Age (Years)	49 (35, 56)	53 (38, 59)	0.006
BMI (kg/m^2^)			0.007
Normal (< 24)	197 (41%)	31 (28%)	
Obesity (24 ~ 28)	88 (18%)	33 (30%)	
Overweight (> 28)	191 (40%)	47 (42%)	
Education level			< 0.001
Junior or below	205 (43%)	43 (39%)	
Secondary	166 (35%)	58 (52%)	
University or above	105 (22%)	10 (9.0%)	
Personnel annual income (CNY)			< 0.001
< 15,000	164 (39%)	18 (18%)	
15,000 ~ 30,000	89 (21%)	30 (29%)	
30,000 ~ 50,000	113 (27%)	26 (25%)	
≥ 50,000	59 (14%)	28 (27%)	
Alcohol drinking (Yes)	31 (6.5%)	49 (44%)	< 0.001
Tea drinking (Yes)	67 (14%)	44 (40%)	< 0.001
Eat before bed			0.058
Never	292 (61%)	78 (70%)	
Only liquid	129 (27%)	18 (16%)	
Some food	55 (12%)	15 (14%)	
Daily tooth brushing frequency			< 0.001
< Once	16 (3.4%)	12 (11%)	
Once	236 (50%)	61 (55%)	
> Once	224 (47%)	38 (34%)	
Average tooth brushing time			0.3
< 3 min	261 (55%)	65 (61%)	
≥ 3 min	210 (45%)	42 (39%)	
Regular oral health screening (Yes)	57 (12%)	17 (16%)	0.3
Chronic disease history (Yes)	147 (31%)	41 (37%)	0.2
Surgical history (Yes)	205 (43%)	44 (40%)	0.5
Antibiotic (Yes)	84 (18%)	15 (14%)	0.3
TG (mmol/L)	1.34 (0.85, 1.94)	1.73 (1.12, 2.45)	< 0.001
CHOL (mmol/L)	4.56 (4.00, 5.13)	4.35 (3.94, 4.93)	0.046
HDL-C (mmol/L)	1.20 (1.07, 1.38)	1.06 (0.91, 1.19)	< 0.001
LDL-C (mmol/L)	2.33 (1.79, 2.82)	2.15 (1.72, 2.54)	0.017
GLU (mmol/L)	5.10 (4.74, 5.53)	5.13 (4.78, 5.51)	0.5
CRP (mg/L)	1.04 (0.82, 1.47)	1.12 (0.93, 1.52)	0.033

Continuous variables were represented as median (IQ1, IQ3). Categorical variables were represented as n (%). The percentage sum of some cells is not equal to 100 because the percentage was rounded to retain decimal places. CNY, China Yuan. BMI, Body mass index. TG, Triglyceride. CHOL, Cholesterol. HDL-C, High-density lipoprotein cholesterol. LDL-C, Low-density lipoprotein cholesterol. GLU, Glucose. CRP, C-reactive protein

### Smoking and oral microbial composition

**Fig. 1:**
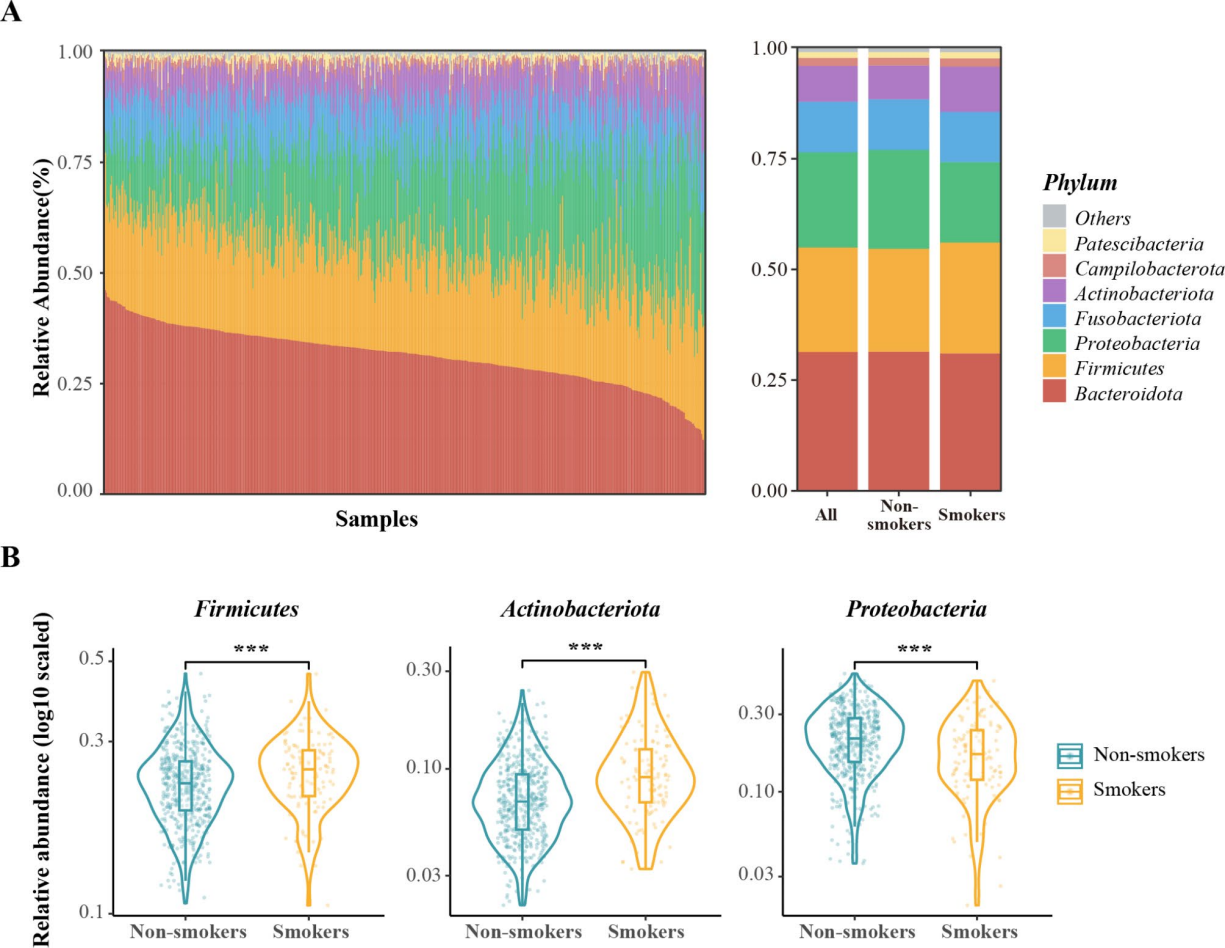
Taxonomic analysis of oral microbiome at phylum level in smokers and non-smokers. (**A**) Taxonomic barplots of the oral microbiome at the phylum level. Taxa with an average relative abundance > 1% were shown, and the rest were grouped into “Others”. The left panels show the results of each individual, whereas the right panels are the averaged values of all samples, smokers and non-smokers. (B) Violin plots of the phyla with significant difference between smokers and non-smokers

The overall oral microbial community at phylum level was shown in Fig. 1A. Seven phyla with 1% higher mean relative abundance were detected, compositing of *Bacteroidota* (31.37%), *Firmicutes* (23.57%), *Proteobacteria* (21.53%), *Fusobacteriota* (11.42%), *Actinobacteriota* (8.10%), *Campilobacterota* (1.78%) and *Patescibacteria* (1.32%) (Fig. 1A). Among them, two phyla *Firmicutes* (average relative abundance: 25.03% vs. 23.22%, *p* < 0.001) and *Actinobacteriota* (average relative abundance: 10.24% vs. 7.61%, *p* < 0.001) were more abundant in smokers, while the relative abundances of *Proteobacteria* (average relative abundance: 18.18% in smokers vs. 22.31% in non-smokers, *p* < 0.001) tended to be less in smokers, compared to that in non-smokers (Fig. 1B).The relative abundance of the remaining major phyla was comparable between two groups (all *p* > 0.05, Supplementary Table S1).

**Fig. 2:**
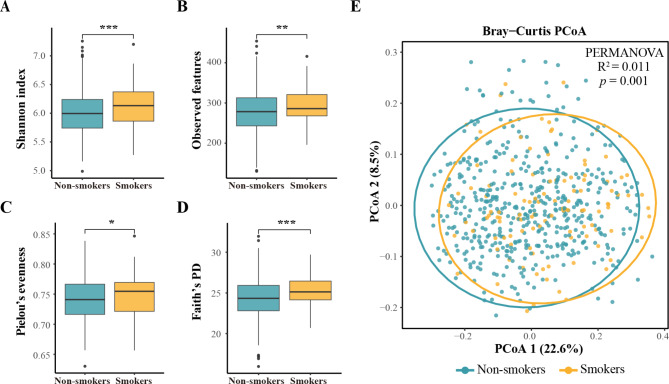
Alpha and beta diversity estimates of the oral microbial community. (**A-D**) Comparison of Shannon index (**A**), Observed features (**B**), Pielou’s evenness (**C**) and Faith’s PD (**D**) in the oral microbiota between smokers and non-smokers. * *p* ≤ 0.05, ** *p* ≤ 0.01, *** *p* ≤ 0.001. (**E**) PCoA based on the Bray-Curtis distances of the oral microbial communities between smokers and non-smokers

In alpha-diversity analysis, different diversity measures for oral bacterial microbiome were used, including Shannon index, Observed features, Faith’s PD, and Pielou’s evenness. These four indices were significantly higher in smokers compared to that in non-smokers (all *p* < 0.05, Fig. 2A-D). For beta-diversity dissimilarity analysis using Bray-Curtis distance matrices, the overall oral microbiota structure significantly differed between two groups (PERMANOVA, *p* < 0.05, Fig. 2E).

Accounting for the potential effect on oral microbial composition interacted between cigarette smoking status and sex, participants were additionally grouped as: male smokers (n = 111), male non-smokers (n = 85), female non-smokers (n = 391). Sensitivity analyses showed that both alpha (Observed features, Shannon and Faith’s PD indices) and beta diversities of oral microbiota were significantly different among three groups (all *p* < 0.05, Supplementary Figure S2). In subgroup analyses, similar results were observed in men between smokers and non-smokers. Though no significant difference in alpha-diversity indices was observed between male and female non-smokers, a modest but statistically significant difference in the overall oral microbiota structure was observed between them (*p* = 0.005, Supplementary Figure S2).

### Smoking, oral microbial taxa, and functional pathways

**Fig. 3:**
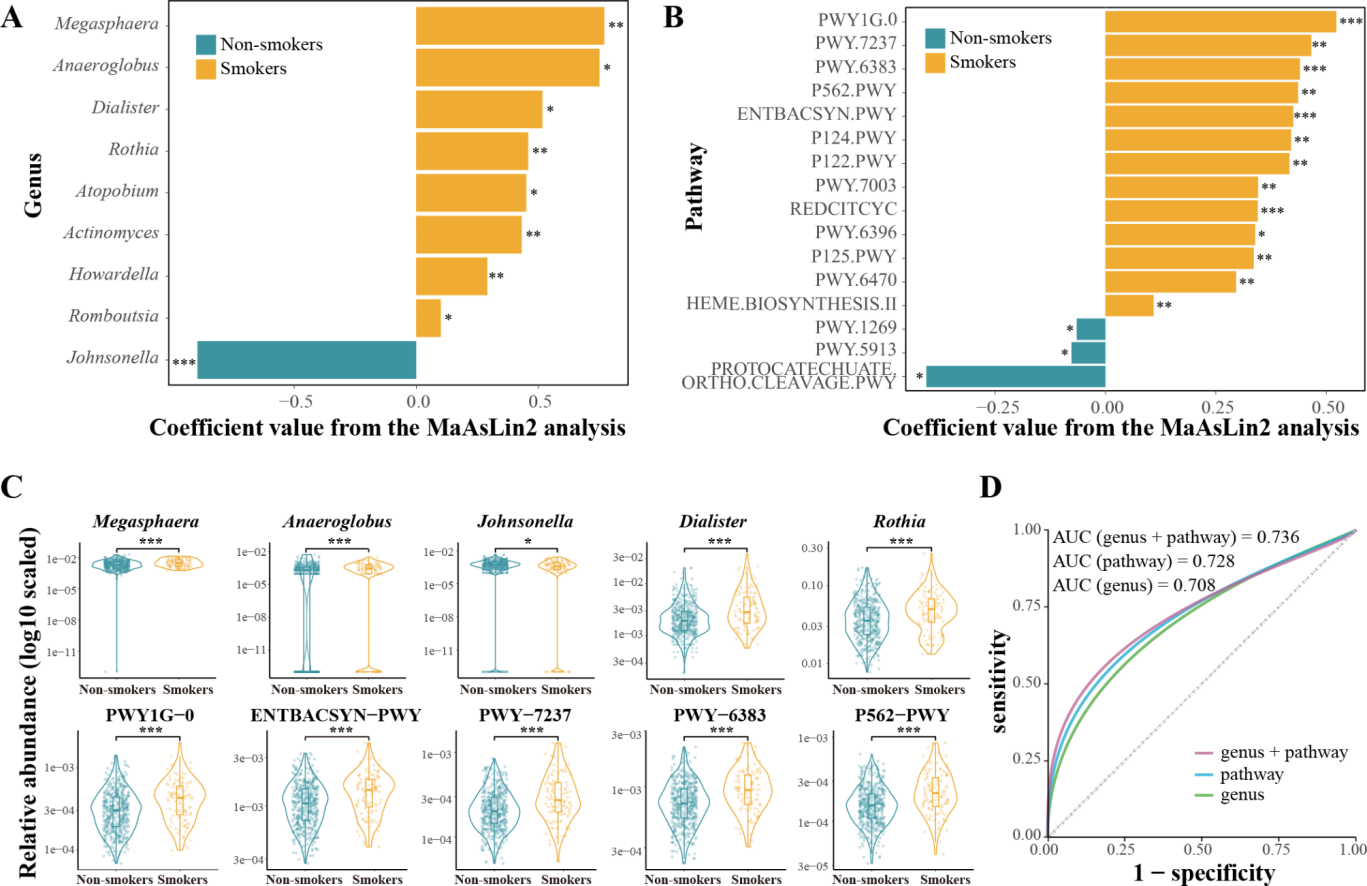
Differences in oral microbiota between smokers and non-smokers and specific bacteria to distinguish smoking status. The result of comparison of bacterial abundance at the genus (**A**) and pathway (**B**) level in smokers and non-smokers from the MaAsLin2 analysis. Adjusted for sex, age, education level, personal annual income, body mass index, alcohol drinking, tea drinking and daily tooth brushing frequency. Results in FDR *q* ≤ 0.10 and absolute coefficient value greater than 0.05 were shown. * FDR *q* ≤ 0.10, ** FDR *q* ≤ 0.05, *** FDR *q* ≤ 0.01. (**C**) The relative abundances of the first five different taxa and pathways were visualized by the violin plots. The x-axis shows cigarette smoking status and y-axis shows the relative abundance. (**D**) ROC curves of oral microbiota in classifying cigarette smoking status

Among 181 common bacterial genera detected in saliva samples, the higher relative abundance of eight genera (i.e., *Megasphaera*, *Anaeroglobus*, *Dialister*, *Rothia*, *Atopobium*, *Actinomyces*, *Howardella*, and *Romboutsia*) and the lower relative abundance of the genus *Johnsonella* in smokers was observed, respectively, compared to that in non-smokers with adjustment for sex, age, education level, personal annual income, BMI, alcohol drinking, tea drinking and daily tooth brushing frequency (FDR *q* < 0.20, Fig. 3A C, and Supplementary Table S2). Specifically, the most notable difference in relative abundance between smokers and non-smokers among these features was observed for *Megasphaera*, with a difference in their median relative abundance of 1.43%. These genera are classified under two phyla, *Firmicutes* and *Actinobacteria*, primarily comprising anaerobic bacteria (Supplementary Table S3). Among 391 functional pathways detected, 26 pathways were identified to be significantly associated with cigarette smoking, with adjustment for the above potential covariates (FDR *q* < 0.20, Fig. 3B C, and Supplementary Table S4).

Based on oral microbial features relating to cigarette smoking, we examined their performance to discriminate cigarette smoking status in bacterial genus or function pathway level alone and in their combination. We observed that nine genera and 26 functional pathways could classify a participant’s cigarette smoking status with an accuracy of 70.8% and 72.8%, respectively. The classification accuracy increased to 73.6% when combined with these identified bacterial genera and pathways (Fig. 3D).

### Microbial features and cardiometabolic risk factors

**Fig. 4:**
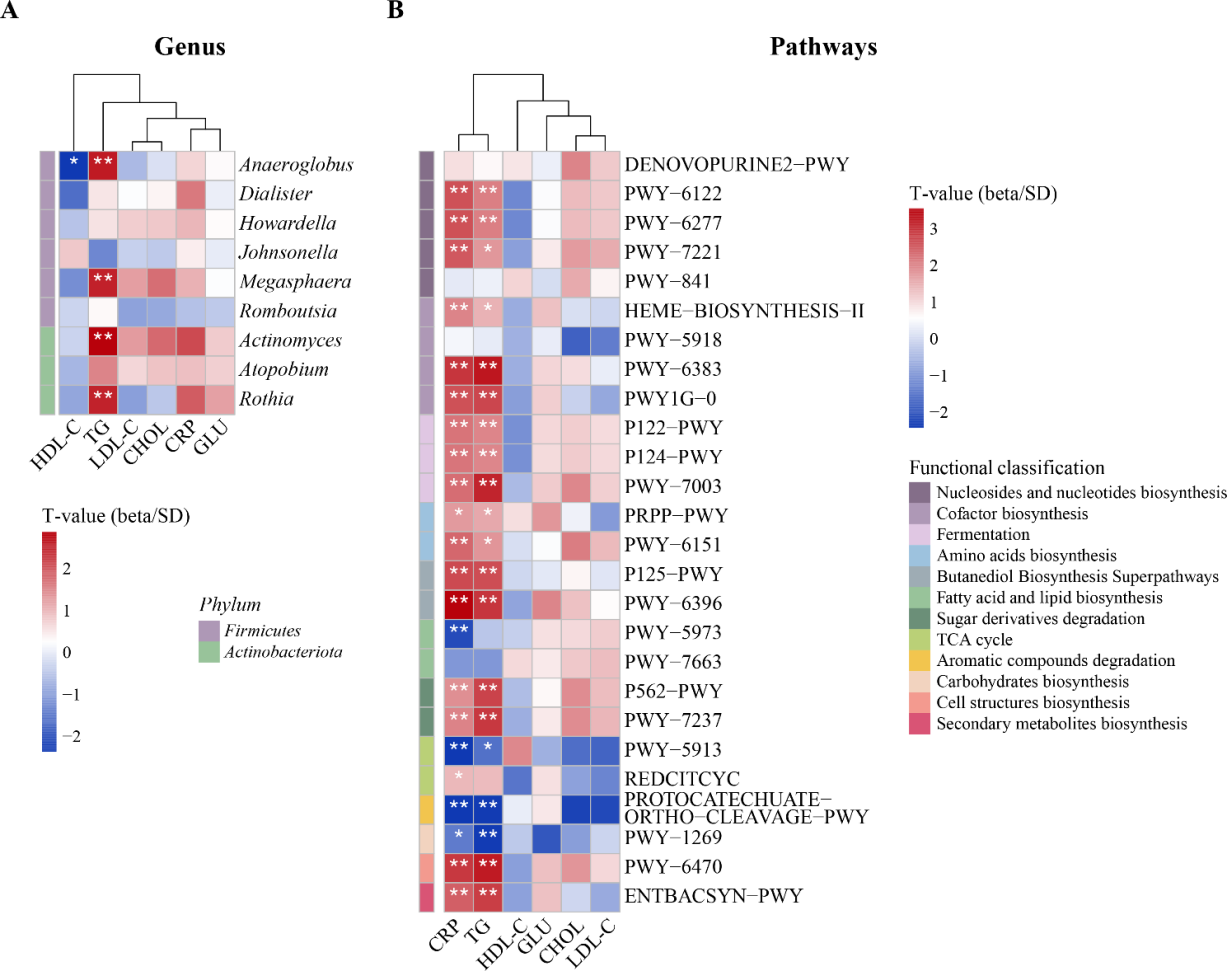
Associations of cigarette smoking-related oral bacterial genera (**A**) and pathways (**B**) with cardiometabolic risk factors. The color of the heat map represents the T value of the relationship between microbial features and cardiometabolic risk factors (that is, the effect value obtained by the regression model divided by SD). * FDR *q* < 0.20, ** FDR *q* < 0.10. Regression models were adjusted for sex, age, education level, personal annual income, body mass index, alcohol drinking, tea drinking and daily tooth brushing frequency. TG, Triglyceride; CHOL, Cholesterol; HDL-C; High-density lipoprotein cholesterol; LDL-C, Low-density lipoprotein cholesterol; GLU, Glucose; CRP, C-reactive protein

After adjusting for sex, age, education level, personal annual income, BMI, alcohol drinking, tea drinking and daily tooth brushing frequency, we observed 47 significant associations (FDR *q* < 0.20) between cigarette smoking-related microbial features and cardiometabolic risk factors (Fig. 4). Specifically, the relative abundance of four bacterial genera (i.e., *Anaeroglobus*, *Megasphaera*, *Actinomyces*, and *Rothia*) out of the eight enriched in smokers were positively associated with the serum TG level (all FDR *q* < 0.20, Fig. 4A), and the relative abundance of the genus *Anaeroglobus* was negatively associated with the serum HDL-C level (FDR *q* = 0.17, Fig. 4A). For identified functional pathways, 20 pathways (e.g., PWY-6383, PWY-6470, and PWY1G-0) and 22 pathways (e.g., PWY-6122, PWY-6277, and PWY-7221) were associated with the serum TG and CRP levels (all FDR *q* < 0.20, Fig. 4B), respectively. Among them, 17 functional pathways exhibited positive associations with serum TG and CRP levels, and these features primarily encompassed processes associated with inositol degradation. Three functional features, negatively correlated with serum TG and CRP levels, were linked to processes involving energy production and conversion, membrane and envelope biogenesis, as well as the degradation of aromatic compounds. The direction of association for the level of cardiometabolic risk factors with these pathways was usually consistent for cigarette smoking status.

**Fig. 5:**
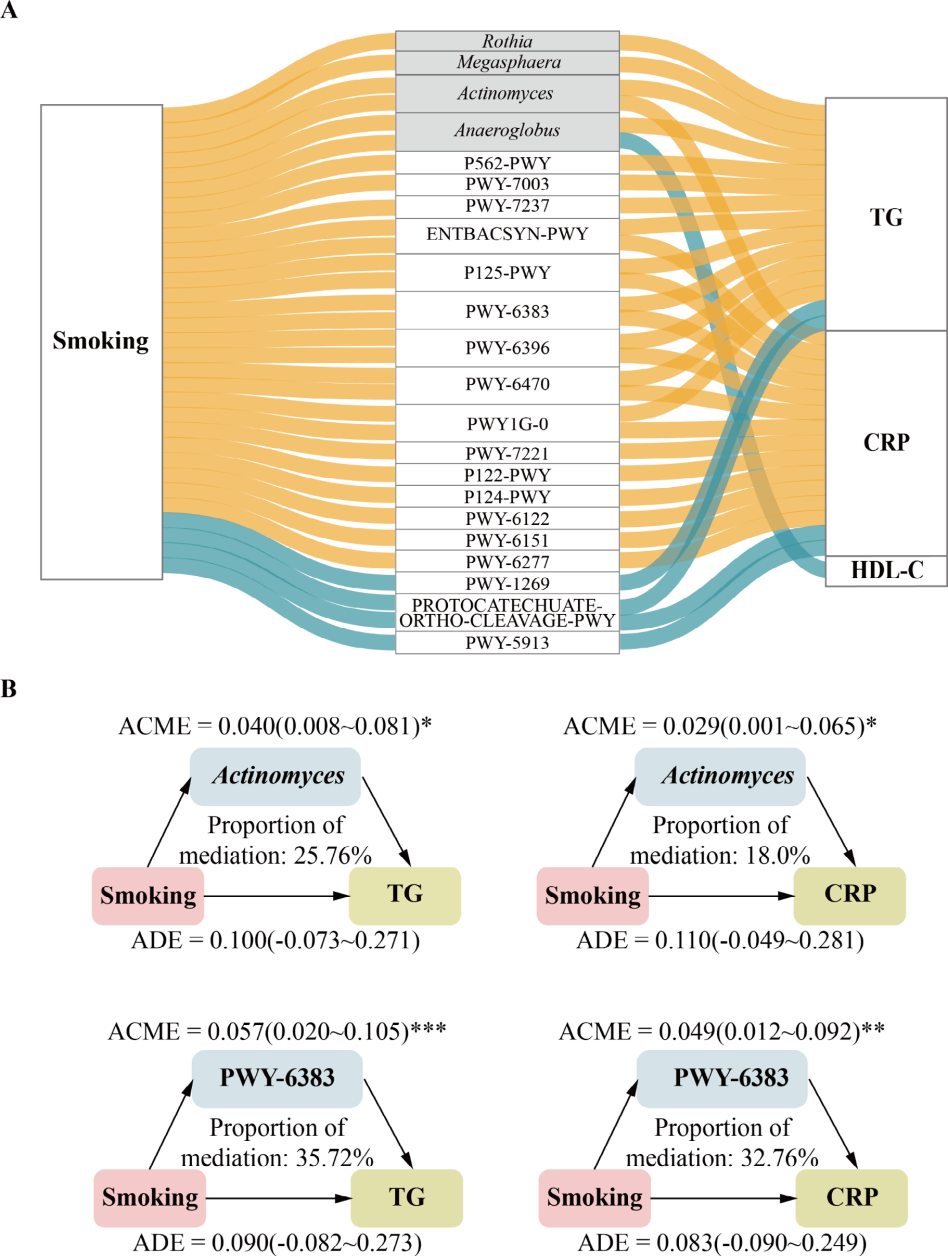
Mediation linkages among cigarette smoking status, oral microbiota, and cardiometabolic risk factors. (**A**) Parallel coordinates chart showing the significant mediation effects of microbial features. The left panel shows cigarette smoking status, the middle panel shows the microbial features, and the right panel shows the cardiometabolic risk factors. Association curves indicate mediation effects. Colors indicate the direction of the association, with blue indicating a negative association and yellow indicating a positive association. The gray background indicates the genus, and the white background indicates the pathway. (**B**) Oral bacterial genera and pathways with the highest proportion of mediators. TG, Triglyceride; CRP, C-reactive protein; HDL-C, High-density lipoprotein cholesterol; ACME, Average causal mediated effect; ADE, Average direct effect; * *p* ≤ 0.05, ** *p* ≤ 0.01, *** *p* ≤ 0.001

In mediation analyses, we evaluated whether the associations of cigarette smoking status with cardiometabolic risk factors were mediated by above-identified microbial features. We observed that 22 microbial features significantly mediated the associations of cigarette smoking with the cardiometabolic risk factors (Fig. 5A and Supplementary Table S5). Specifically, the associations between cigarette smoking and serum TG level were mediated by the relative abundance of oral bacterial genera *Actinomyces*, *Megasphaera*, *Rothia*, and *Anaeroglobus*, with the contribution proportion of 25.76%, 20.89%, 20.31%, and 17.95%, respectively (all *p*-mediation < 0.05). The genera *Anaeroglobus* (contribution proportion: 20.74%) and *Actinomyces* (contribution proportion: 18.01%) also contributed to the associations of cigarette smoking with serum HDL-C and CRP levels, respectively. For functional features, 18 pathways were observed to significantly mediate the associations of cigarette smoking with serum TG and CRP levels (all *p*-mediation < 0.05), with contribution proportions ranging from 35.72% for PWY-6383 to 15.51% for PROTOCATECHUATE-ORTHO-CLEAVAGE-PWY. The bacteria genera and pathways with the highest mediating proportion was *Actinomyces* and PWY-6383, respectively, and them both mediated the associations of cigarette smoking with serum TG and CRP levels (Fig. 5B).

We further examined the associations of the pack-years of cigarette smoking with microbial mediators. The pack-years were positively associated with three bacteria mediators (i.e., *Megasphaera*, *Rothia*, and *Anaeroglobus*) and ten functional mediators (e.g., PWY-6277, PWY-6383, and PWY1G-0). In contrast, inverse associations were observed between the pack-years and PROTOCATECHUATE-ORTHO-CLEAVAGE-PWY and PWY-1269 (FDR *q* < 0.20, Supplementary Figure S3).

## Discussion

In the present study, we observed a modest but statistically significant difference in the oral microbial composition between smokers and non-smokers. Distinct bacterial taxonomic and functional pathways were observed between the two groups, including an increase in the relative abundance of eight genera (e.g., *Megasphaera*) and 21 pathways (e.g., PWY-7237 and P562-PWY) enriched in smokers in comparison of that of non-smokers. Some cigarette smoking-associated microbial features partly mediated the disparities of cardiometabolic risk factors according to cigarette smoking status, indicating that oral microbiota may play a role in the potential impact of cigarette smoking on cardiometabolic health.

Our study demonstrated varied microbial diversity and overall community structure according to cigarette smoking status, based on saliva samples from Chinese adults. These observations were consistent with findings from the United Arab Emirates Healthy Future Study conducted among 105 smokers and 225 non-smokers using mouth wash samples [10], though its Shannon indices (median values ranging from 3.0 to 3.5) were higher than that in our study (median values ranging from 6.0 to 6.5). The study also indicated that smokers had higher alpha diversity of the oral microbiota compared to that of non-smokers, and the overall microbiota structure differed between the two groups, despite diverse methods of sample collection and types of cigarette products consumed by different populations. In two population-based studies of American adults [7, 8] using more than 1,200 mouth rinse samples, the significant difference in oral microbiome composition between current and non-current smokers were presented as well. In contrast, Yu and colleagues did not find any significant difference in microbial diversity and composition in any of eight oral sites (including saliva and mouth wash samples) between 23 current smokers and 20 never smokers [9]. These inconsistent observations may be attributable to sample sizes and multiple influential factors including ethnicity, diet, and environment [12]. Nonetheless, various studies have provided support for discrepancies in the oral microbiota community according to cigarette smoking status. Its biological implications should be interpreted with caution, given the large range of oral microbial diversity and the inconsistencies observed among previous studies.

The present study characterized a panel of oral microbes that were associated with cigarette smoking in Chinese adults. The phyla *Firmicutes* and *Actinobacteriota* were related to cigarette smoking, which is consistent with the observation in previous studies [8, 13, 14]. Within the two phyla, most detected bacterial genera with an increased relative abundance in smokers were anaerobic (e.g., *Megasphaera*, *Anaeroglobus*, and *Dialister)* or facultative anaerobic (i.e., *Actinomyces)*, which may be induced by the oxygen-deprived environment in oral cavity due to the exposure to cigarette smoke [15]. Three (i.e., *Megasphaera*, *Actinomyces*, and *Atopobium)* of these differential genera were also more abundant in smokers than that of never smokers in a Chinese population with a sample size of 316 participants [16]. In addition, *Dialister* and *Rothia* were commonly detected in oral samples such as tongue, tonsils, and saliva, and their abundances also have been positively linked to cigarette smoking’s pack-years or duration [8, 17–21].These differentially abundant microbes suggested to increase the risks of periodontal and systematic diseases [3, 4]. For example, *Dialister* and *Atopobium* were both identified as periodontopathogens bacteria [22, 23], and the abundance of *Megasphaera* and *Rothia* was increased in patients with oral ulcers [21] and stomatitis [24] when compared to that of healthy controls.

Consistent with previous observations, most oral microbial functions associated with cigarette smoking in the present study were linked to diverse anaerobic fermentation pathways for carbohydrate and pyruvate [16, 17]. This may be partially related to the predominance of anaerobic bacteria in the oral microbiota of smokers. For lactate, acetate, ethanol, and formate produced via these fermented pathways, close associations of these acidic by-products with periodontitis and dental health have been illustrated [5, 25]. The enrichment of acid products may cause the reductions on salivary pH, which increased the demineralization of dental enamel and the production of biofilm exopolysaccharide matrix [5]. Within this biofilm, microorganisms continue to generate acid and entrap it on the enamel surfaces, further expediting the demineralization process of dental enamel while simultaneously raising the acidity level in the oral environment [26]. These alterations can disrupt the delicate balance of oral pH, subsequently stimulating the oral mucosa and gingiva. This stimulation results in heightened cellular membrane permeability and an inflammatory response, consequently impacting oral health [27]. Furthermore, we found that cigarette smokers also had active pathways involving mycothiol (PWY1G-0), enterobactin (ENTBACSYN-PWY), and peptidoglycan (PWY-6470) biosynthesis. Mycothiol is a reducing agent with glutathione-like function [28], which participated in the higher level of oxidative stress caused by cigarette smoke [29]. Some products (e.g., enterobactin and peptidoglycan) generated from these pathways were reported to correlate with enhancement of oral microbial toxicity [30, 31].

Our results suggest that some oral microbial features associated with cigarette smoking may potentially interplay with cardiometabolic health, especially in lipid metabolism and inflammatory response. Several specific oral microbes and functional pathways have been linked to the metabolic phenotypes. For example, the genera *Megasphaera* [32, 33] and *Anaeroglobus* [34] was reported to be enriched in the oral microbiota of overweight children and patients with atherosclerosis, respectively. In vitro experiments, lipopolysaccharide, a kind of cell wall product released by oral bacteria, was indicated to increase the lipolytic activity of adipocytes which can subsequently increase serum TG level [35]. Inositol reduced by the inositol degradation pathway (PWY-7237 and P562-PWY) has been found to protect against hepatic steatosis [36]. In terms of inflammatory response, studies demonstrated that toxicants generated from cigarette smoking can facilitate the adhesion and colonization of periodontal pathogens and promote the delivery of pro-inflammatory cytokines into the host circulation, the enrichment of these microorganisms, thus, can induce local and systematic inflammation [37]. Common inflammatory phenotypes in oral (e.g., periodontitis) can stimulate the increase in circulating inflammatory factors such as CRP, Interleukin-1, Interleukin-6, and Interleukin-8 as well [38, 39]. Adenosine and prenylated proteins were important regulators in identified pathways for purine nucleotides synthesis (PWY-6122, PWY-6277, and PWY-7221) and for decaprenyl phosphate biosynthesis (PWY-6383), respectively, and they have been implicated in diseases ranging from inflammation to cancer [40, 41].

The present study was limited by several factors. First, 16S rRNA gene sequencing was used to assess the oral microbiome, which may not be precise enough for assessing the species level microbial profile and microbial pathways. Considering that PICRUSt2 generates a “predicted” functional activity, rather than a measured function, it is crucial to exercise caution during interpretation. And further verification through metagenomic analysis and functional experiments in future research are essential for a more precise understanding of the microbial community structure and its functions. Second, the cigarette smoking status was assessed by questionnaires, which are subject to the influence of recall bias. The potential measurement error and confounders may still be a concern. Third, the cross-sectional nature of our study does not allow us to draw any causal conclusions. Additionally, all smokers were male and participants included residents from the central plain of China, impairing the ability to generalize the results to the Chinese population in part.

## Conclusions

In summary, this study showed distinct differences in oral microbial diversity and overall structure, individual taxonomic and functional features, according to cigarette smoking status in a Chinese population. The identified features of the oral microbiota may partially mediate the associations of cigarette smoking and cardiometabolic risk factors. Large prospective studies using the shotgun metagenomic sequencing technique are needed to further examine the oral microbiome effect produced by cigarette smoking.

## Materials and methods

### Populations and study design

The present study was based on data from the Central China Cohort (CCC), an ongoing population-based study conducted in Xinmi city, Henan province of China. The CCC was initiated in 2017 and designed to achieve a comprehensively phenotypic measurement of natives at both macro and molecular levels to explore associations of human physique, structure, and functions with common diseases. At enrollment, participants were interviewed to collect their information on sociodemographics, diets/lifestyles, disease and medical history, and anthropometrics; blood specimens were also collected. During the first follow-up visit in 2020, saliva and stool samples for multiomics assessments were also collected. Starting with 612 participants recruited in 2020, we then excluded participants without detailed information on cigarette smoking status (n = 7), saliva samples (n = 10), or with < 10,000 sequence reads in their saliva samples (n = 8), thus remaining 587 participants in the analysis of the associations of the oral microbiome with cigarette smoking status. Two participants without blood samples were further excluded in analysis of the identified microbial features with the cardiometabolic risk factors. All participants signed written informed consent forms.

### Smoking and covariate collection

Physical measurements (e.g., height, weight, and waist circumference) and standard questionnaire interview were conducted by trained health investigators, covering sociodemographic status (e.g., sex, age, and education), lifestyle (e.g., smoking, alcohol, and tea consumption), and medical information (diseases history, drug history and antibiotic use) during each survey period. Questions on smoking status included smoking status (current, former, or never smoking); and for smokers, age when individuals first began to smoke regularly, duration, frequency, dose (pack-years) of cigarette smoking, and degree of inhalation. Participants who smoked one or more cigarettes every three days for at least 6 consecutive months were classified as smokers, while those who had not were considered non-smokers. For the smoking intensity in smokers, pack-years were calculated by multiplying the reported average packs of cigarettes smoked per day (duration over which this average was estimated) by the number of years of smoking [42].

### Saliva sample collection and microbiota profiling

During the follow-up visit at the study site, saliva samples (2 mL in a funnel-type collection tube, without foam) were collected by the participants themselves, who received instructions for the collection process, and immediately stored at 4 °C fridge. All saliva samples were transported to the research laboratory on dry ice within 24 h and divided into aliquots and stored in − 80 °C freezers until processing.

Details on oral microbial DNA extraction and Illumina sequencing library preparation were described previously [43]. Briefly, microbial genomic DNA was extracted using the DNeasy^®^ 96 PowerSoil^®^ Pro QIAcube^®^ HT Kit (Qiagen, Germany) and purified with the QIAcube HT system (Qiagen, Germany) according to the manufacturer’s recommendations. Primers 515F/806R (5’-barcode-GTGYCAGCMGCCGCGGTAA-3’/ 5’-GGACTACNVGGGTWTCTAAT-3’) [44] were used to amplify the V4 region of 16S rRNA gene with a 12-bp barcode unique to each sample. Illumina sequencing libraries were prepared via KAPA LTP Library Preparation Kit (KK8233, Roche, Switzerland) and PKR Y Type Adapter Kit (Pukairui, China) according to manufacturer’s instructions. The PCR products were purified using AxyPrep DNA Gel Extraction Kit (Axygen, USA) and quantified using the Qubit 4 Fluorometer (Thermo Fisher Scientific, USA). Pooled amplicon libraries were sequenced on the Illumina NovaSeq 6000 platform using 2 × 250 bp paired-end strategy. The subsequence amplicon sequence analysis was performed with QIIME2 version 2022.2.0 [45]. After demultiplexing, paired-end sequencing reads were quality-filtered, trimmed, denoised, and merged using DADA2 pipeline in QIIME2 (trim-left-f = 19, trim-left-r = 20, trunc-len-f = 160, trunc-len-r = 160, max-ee = 2, trunc-q = 2) [46]. Reads were then summarized to amplicon sequence variant (ASV) in a feature table and annotated using the Naïve Bayes classifier trained on the SILVA 138 SSU Ref NR 99 data set [47]. ASVs present in only one sample or with total abundances < 10 were excluded using the q2-feature-table filter. Based on rarefaction curve analysis (Figure **S1**), four alpha-diversity indices were calculated at the sampling depth of 10,000: Shannon diversity, Observed features, Pielou’s evenness and Faith’s phylogenetic diversity (PD). We performed functional prediction from the ASV table using the PICRUSt2 algorithm [48].

### Measurement of cardiometabolic risk factors

Fasting blood samples after an overnight fast of at least 8 h were collected in 2020. Serum high-density lipoprotein cholesterol (HDL-C), low-density lipoprotein cholesterol (LDL-C), total cholesterol (TC), and triglycerides (TG) were measured using the enzymatic method. Serum glucose (GLU) and C-reactive protein (CRP) was measured by the hexokinase method and turbidimetric immunoassay, respectively. These cardiometabolic risk factors were measured on the automatic analyzer (Olympus AU400, Japan).

### Statistical analysis

Difference in participant characteristics between smokers and non-smokers was tested using Student’s t-test and Chi-square test for continuous variables and categorical data, respectively. Wilcoxon-Mann-Whitney test was performed to examine the differences in taxonomic phylum features and four alpha-diversity indices between two groups. We calculated the Bray–Curtis dissimilarity metrics for each sample using taxonomic data at ASV level, and then performed permutational multivariate analysis of variance (PERMANOVA) to assess the associations between cigarette smoking status and overall microbial structure. The distance matrix was visualized through principal coordinates analysis (PCoA). Analyses were performed with *vegdist*, *cmdscale* and *adonis2* function of the *vegan* R package (version: 2.6-4). For individual taxa and function analysis, we filtered out all genera and pathways with a prevalence of less than 0.01 followed by LOG transformation. We used Multivariate Analysis by Linear Models (MaAsLin) to identify potential features associated with cigarette smoking, adjusted for sex, age, education level, personal annual income, body mass index (BMI), alcohol drinking, tea drinking and daily tooth brushing frequency. Multiple comparisons were controlled by false discovery rate (FDR). An FDR value < 0.20 was considered statistically significant. We assessed the ability of identified microbial features to classify cigarette smoking status using random forest models. The performance of the models was quantified with receiver operating characteristic (ROC) area under the curve (AUC). Multivariate linear regression models were used to examine the associations of identified microbial features and pack-years of cigarette smoking with the cardiometabolic risk factors, adjusted for the same covariates as above MaAsLin analysis. In addition, we used mediation analyses to investigate the associations among cigarette smoking status, identified microbial features, and cardiometabolic risk factors. We constructed two linear regression models to regress the outcome (cardiometabolic risk factors) on the exposure (smoking status) and mediators (identified microbial features), with adjustment of potential confounders. We integrated these two regressions to obtain the estimates for direct and indirect effects using the regression-based approach. The mediation analysis was performed using the *mediation* R package. We used R version 4.1.2 for statistical analysis, and *p* value < 0.05 was considered statistically significant unless otherwise specified.

### Electronic supplementary material

Below is the link to the electronic supplementary material.

Supplementary Material 1

## Data Availability

Sequencing data during the current study can be viewed in NODE database (https://www.biosino.org/node/project/detail/OEP004135) and are available upon acceptance of the publication.
