# Supplementary material for: Association of cigarette smoking with oral bacterial microbiota and cardiometabolic health in Chinese adults
Source: BMC Microbiol. 2023 Nov 17;23:346. doi: 10.1186/s12866-023-03061-y (PMC10655299; doi:10.1186/s12866-023-03061-y)
Supplement: Supplementary file 1 — Supplementary Material 1 [file 12866_2023_3061_MOESM1_ESM.docx]

**Supplementary Materials**

**Figure S1:** Rarefaction curves of Shannon index (A) and Observed features (B) of oral microbiota; **Figure S2:** Alpha and beta diversity estimates of the oral microbial community;

**Figure S3:** Association of the pack-years of cigarette smoking with microbial mediators;

**Table S1:** Phylum-level composition of the oral microbiome by cigarette smoking status;

**Table S2:** Bacterial genera significantly associated with cigarette smoking status identified by MaAsLin2;

**Table S3.** The median relative abundance of differential genus features by cigarette smoking status.

**Table S4:** Functional pathways significantly associated with cigarette smoking status identified by MaAsLin2;

**Table S5:** Mediation linkages among cigarette smoking status, oral microbiota, and cardiometabolic risk factors.


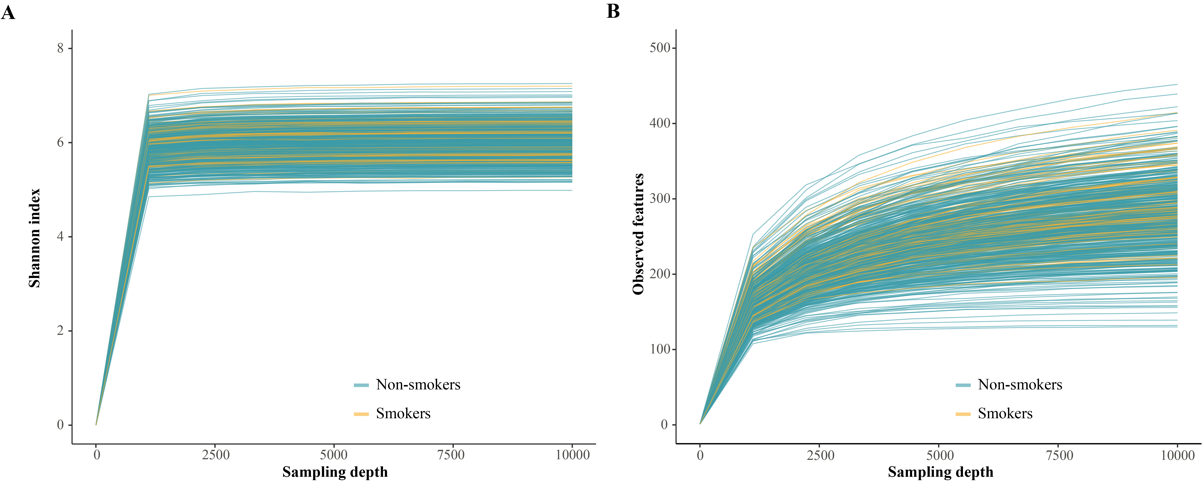


**Figure S1.** Rarefaction curves of Shannon index (**A**) and Observed features (**B**) of oral microbiota.


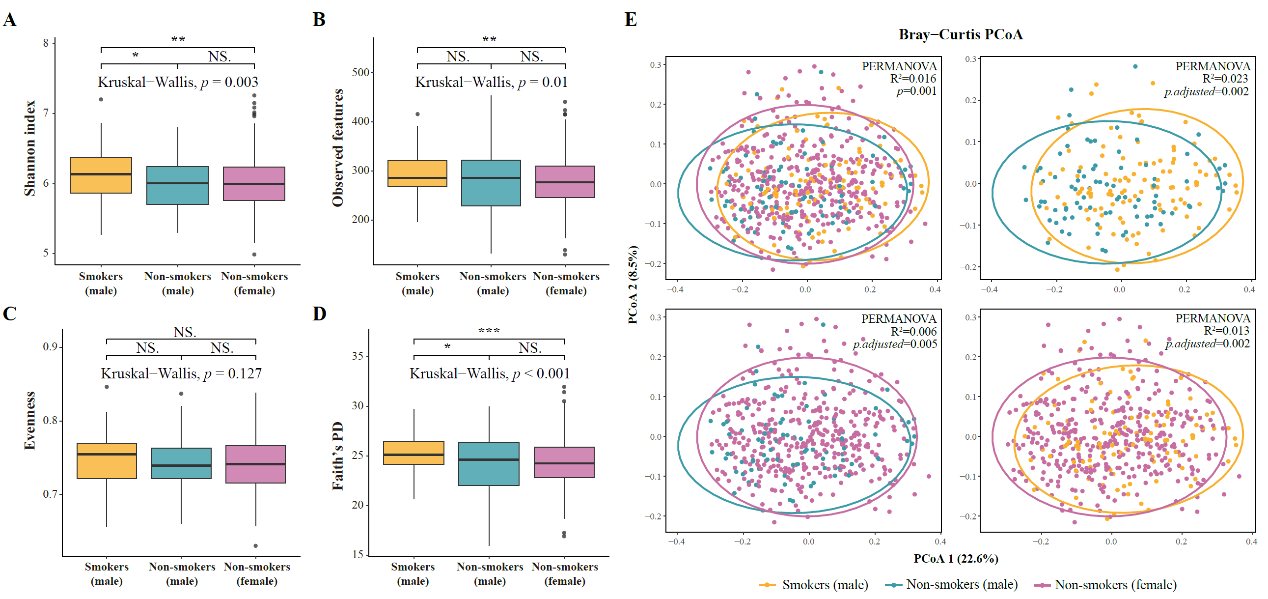
**Figure S2.** Alpha and beta diversity estimates of the oral microbial community. Comparison of Shannon index (**A**), Observed features (**B**), Pielou’s evenness (**C**) and Faith’s PD (**D**) in the oral microbiota between male smokers, male non-smokers, and female non-smokers. * *p* ≤ 0.05, ** *p* ≤ 0.01, *** *p* ≤ 0.001, NS, Non-significant. (**E**) PCoA based on the Bray-Curtis distances of the oral microbial communities among male smokers, male non-smokers, and female non-smokers. *p*.*adjusted* values were adjusted using the Benjamini-Hochberg method.


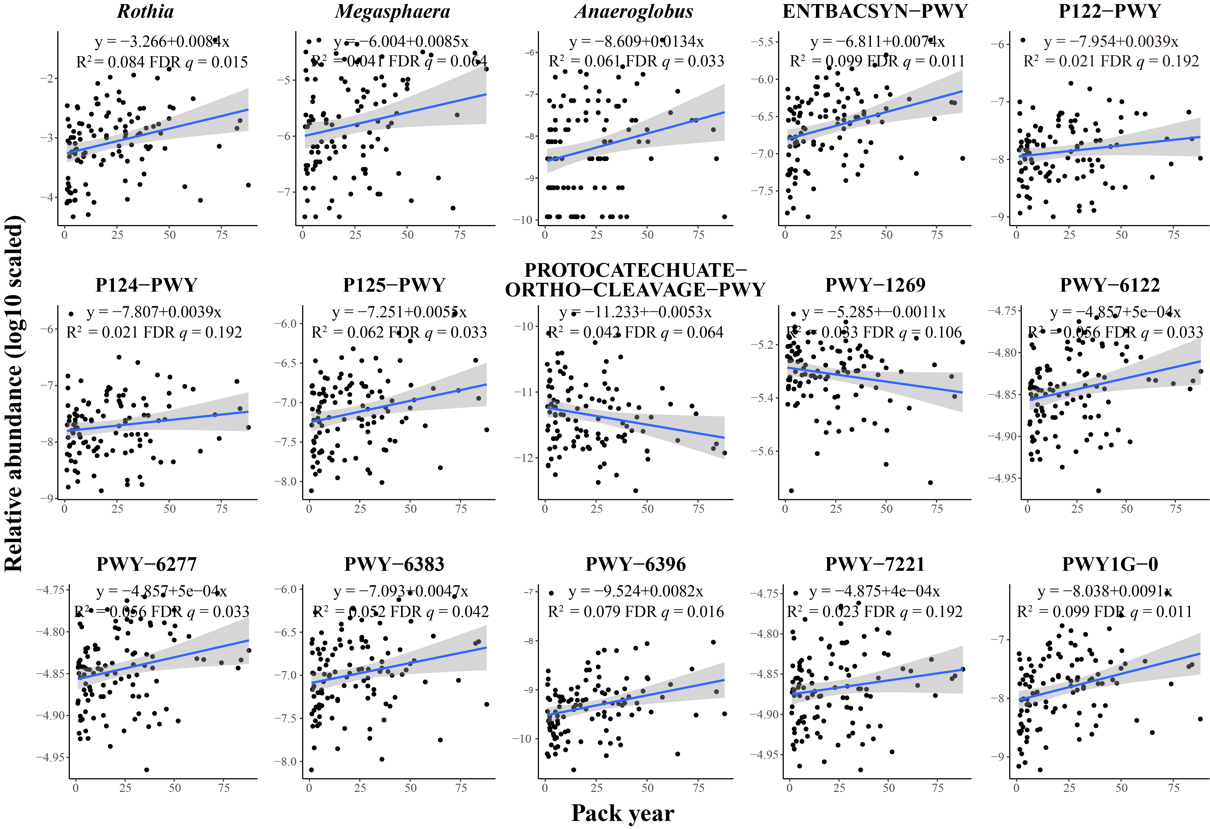


**Figure S3.** Association of the pack-years of cigarette smoking with microbial mediators. R^2^ refers to the explanatory degree of the change in the relative abundance of oral microbial characteristics (log transformed) by pack-years of smoking. Only FDR *q* < 2.0 were shown.

**Table S1.** Phylum-level composition of the oral microbiome by cigarette smoking status.

| ***Phylum*** | **Mean relative abundance** | | | ***p* value** |
| --- | --- | --- | --- | --- |
|  | **ALL (N=587)** | **Non-smoker (N=476)** | **Smoker (N=111)** |  |
| *Bacteroidota* | 31.37% | 31.45% | 31.04% | 0.599 |
| *Firmicutes* | 23.57% | 23.22% | 25.03% | <0.001 |
| *Proteobacteria* | 21.53% | 22.31% | 18.18% | <0.001 |
| *Fusobacteriota* | 11.42% | 11.45% | 11.32% | 0.912 |
| *Actinobacteriota* | 8.10% | 7.61% | 10.24% | <0.001 |
| *Campilobacterota* | 1.78% | 1.76% | 1.86% | 0.180 |
| *Patescibacteria* | 1.32% | 1.31% | 1.40% | 0.241 |
| *Others* | 0.90% | 0.90% | 0.92% | 0.305 |

Taxa with a mean relative abundance >1% were shown, and the rest were grouped into "*Others*". *p* values were from Wilcoxon rank sum test.

**Table S2.** Bacterial genera significantly associated with cigarette smoking status identified by MaAsLin2.

| ***genus*** | **Coef** | **FDR *q*** |
| --- | --- | --- |
| *Megasphaera* | 0.782 | 0.013 |
| *Anaeroglobus* | 0.766 | 0.074 |
| *Dialister* | 0.514 | 0.071 |
| *Rothia* | 0.459 | 0.018 |
| *Atopobium* | 0.455 | 0.078 |
| *Actinomyces* | 0.437 | 0.013 |
| *Howardella* | 0.297 | 0.020 |
| *Romboutsia* | 0.100 | 0.054 |
| *Johnsonella* | -0.897 | 0.004 |

Coef is the effect value obtained from MaAsLin2. MaAsLin, Multivariate Analysis by Linear Models.

**Table S3.** The median relative abundance of differential genus features by cigarette smoking status.

| ***genus*** | ***phylum*** | ***family*** | **Non-smoker** | **Smoker** | ***p* value** |
| --- | --- | --- | --- | --- | --- |
| *Megasphaera ^a^* | *Firmicutes* | *Veillonellaceae* | 0.0355 | 0.0498 | < 0.001 |
| *Anaeroglobus ^a^* | *Firmicutes* | *Veillonellaceae* | 0.0203 | 0.0282 | < 0.001 |
| *Dialister ^a^* | *Firmicutes* | *Veillonellaceae* | 0.0037 | 0.0051 | < 0.001 |
| *Rothia ^c^* | *Actinobacteriota* | *Micrococcaceae* | 0.0020 | 0.0031 | < 0.001 |
| *Atopobium ^a^* | *Actinobacteriota* | *Atopobiaceae* | 0.0020 | 0.0028 | < 0.001 |
| *Actinomyces ^b^* | *Actinobacteriota* | *Actinomycetaceae* | 0.0005 | 0.0004 | < 0.001 |
| *Howardella ^a^* | *Firmicutes* | *Lachnospiraceae* | 0.0002 | 0.0003 | < 0.001 |
| *Romboutsia ^a^* | *Firmicutes* | *Peptostreptococcaceae* | 1E-13 | 1E-13 | 0.005 |
| *Johnsonella ^a^* | *Firmicutes* | *Lachnospiraceae* | 1E-13 | 1E-13 | 0.035 |
| Data were represented as median. Wilcoxon-Mann-Whitney test was performed to examine the differences in the relative abundance of genus features between two groups.  ^a^ anaerobic bacteria, ^b^ facultative bacteria, ^c^ aerobic bacteria. | | | | | |

**Table S4.** Functional pathways significantly associated with cigarette smoking status identified by MaAsLin2.

| **Functional pathway** | **Full name** | **Class name** | **Coef** | **FDR *q*** |
| --- | --- | --- | --- | --- |
| PWY1G-0 | mycothiol biosynthesis | cofactor biosynthesis | 0.523 | 0.003 |
| PWY-7237 | myo-, chiro- and scillo-inositol degradation | sugar derivatives degradation | 0.466 | 0.014 |
| PWY-6383 | mono-trans, poly-cis decaprenyl phosphate biosynthesis | cofactor biosynthesis | 0.440 | 0.001 |
| P562-PWY | myo-inositol degradation I | sugar derivatives degradation | 0.436 | 0.017 |
| ENTBACSYN-PWY | enterobactin biosynthesis | secondary metabolites biosynthesis | 0.425 | 0.005 |
| P124-PWY | Bifidobacterium shunt | carbohydrates degradation | 0.420 | 0.017 |
| P122-PWY | heterolactic fermentation | fermentation | 0.416 | 0.017 |
| PWY-7003 | glycerol degradation to butanol | secondary metabolites biosynthesis | 0.345 | 0.010 |
| REDCITCYC | TCA cycle VIII (helicobacter) | TCA cycle | 0.345 | 0.002 |
| PWY-6396 | superpathway of 2,3-butanediol biosynthesis | fermentation | 0.339 | 0.071 |
| P125-PWY | superpathway of (R,R)-butanediol biosynthesis | fermentation | 0.336 | 0.023 |
| PWY-6470 | peptidoglycan biosynthesis V (beta;-lactam resistance) | cell structures biosynthesis | 0.296 | 0.026 |
| HEME-BIOSYNTHESIS-II | heme biosynthesis I (aerobic) | cofactor biosynthesis | 0.109 | 0.015 |
| PWY-5918 | superpathay of heme biosynthesis from glutamate | cofactor biosynthesis | 0.050 | 0.098 |
| PWY-6151 | S-adenosyl-L-methionine cycle I | amino acids biosynthesis | 0.038 | 0.097 |
| PRPP-PWY | superpathway of histidine, purine, and pyrimidine biosynthesis | amino acids biosynthesis | 0.032 | 0.016 |
| PWY-6122 | 5-aminoimidazole ribonucleotide biosynthesis II | nucleosides and nucleotides biosynthesis | 0.030 | 0.071 |
| PWY-6277 | superpathway of 5-aminoimidazole ribonucleotide biosynthesis | nucleosides and nucleotides biosynthesis | 0.030 | 0.071 |
| PWY-7221 | guanosine ribonucleotides de novo biosynthesis | nucleosides and nucleotides biosynthesis | 0.029 | 0.088 |
| DENOVOPURINE2-PWY | superpathway of purine nucleotides de novo biosynthesis II | nucleosides and nucleotides biosynthesis | 0.024 | 0.070 |
| PWY-841 | superpathway of purine nucleotides de novo biosynthesis I | nucleosides and nucleotides biosynthesis | 0.022 | 0.073 |
| PWY-7663 | gondoate biosynthesis (anaerobic) | fatty acid and lipid biosynthesis | -0.024 | 0.099 |
| PWY-5973 | cis-vaccenate biosynthesis | fatty acid and lipid biosynthesis | -0.026 | 0.023 |
| PWY-1269 | CMP-3-deoxy-D-manno-octulosonate biosynthesis | carbohydrates biosynthesis | -0.065 | 0.082 |
| PWY-5913 | partial TCA cycle (obligate autotrophs) | TCA cycle | -0.077 | 0.099 |
| PROTOCATECHUATE-ORTHO-CLEAVAGE-PWY | protocatechuate degradation II (ortho-cleavage pathway) | aromatic compounds degradation | -0.405 | 0.081 |

Coef is the effect value obtained from MaAsLin2. MaAsLin, Multivariate Analysis by Linear Models.

**Table S5.** Mediation linkages among cigarette smoking status, oral microbiota, and cardiometabolic risk factors.

| **Mediator (Microbial features)** | **Outcome (Serum metabolic biomarkers)** | **ACME** | **ADE** | **TE** | **Contribution proportion** | ***p*-mediation** |
| --- | --- | --- | --- | --- | --- | --- |
| *Rothia* | TG | 0.033(0.003~0.071) * | 0.110(-0.054~0.275) | 0.143(-0.021~0.309) | 20.31% | 0.03 |
| *Actinomyces* | TG | 0.040(0.008~0.081) * | 0.100(-0.073~0.271) | 0.140(-0.030~0.311) | 25.76% | 0.016 |
| *Actinomyces* | CRP | 0.029(0.001~0.065) * | 0.110(-0.049~0.281) | 0.138(-0.018~0.312) | 18.01% | 0.044 |
| *Megasphaera* | TG | 0.034(0.005~0.072) * | 0.112(-0.071~0.291) | 0.146(-0.032~0.325) | 20.89% | 0.018 |
| *Anaeroglobus* | HDL-C | -0.010(-0.023~-0.001) * | -0.022(-0.088~0.042) | -0.032(-0.095~0.034) | 20.74% | 0.036 |
| *Anaeroglobus* | TG | 0.030(0.003~0.066) * | 0.114(-0.048~0.284) | 0.144(-0.021~0.315) | 17.95% | 0.018 |
| ENTBACSYN-PWY | TG | 0.043(0.010~0.082) ** | 0.100(-0.063~0.280) | 0.143(-0.025~0.325) | 27.90% | 0.01 |
| ENTBACSYN-PWY | CRP | 0.036(0.003~0.076) * | 0.105(-0.076~0.278) | 0.141(-0.043~0.314) | 22.60% | 0.036 |
| P122-PWY | CRP | 0.028(0.001~0.066) * | 0.108(-0.057~0.285) | 0.137(-0.025~0.308) | 18.68% | 0.044 |
| P124-PWY | CRP | 0.029(0.002~0.068) * | 0.107(-0.058~0.269) | 0.136(-0.023~0.293) | 18.38% | 0.036 |
| P125-PWY | TG | 0.034(0.004~0.075) * | 0.105(-0.070~0.281) | 0.139(-0.038~0.310) | 21.83% | 0.012 |
| P125-PWY | CRP | 0.034(0.007~0.070) * | 0.102(-0.078~0.274) | 0.136(-0.035~0.300) | 20.77% | 0.012 |
| P562-PWY | TG | 0.037(0.007~0.076) * | 0.104(-0.077~0.274) | 0.141(-0.038~0.325) | 22.82% | 0.014 |
| PROTOCATECHUATE-ORTHO-CLEAVAGE-PWY | CRP | 0.025(0.002~0.057) * | 0.106(-0.075~0.274) | 0.131(-0.042~0.305) | 16.33% | 0.038 |
| PROTOCATECHUATE-ORTHO-CLEAVAGE-PWY | TG | 0.025(0.002~0.063) * | 0.116(-0.052~0.285) | 0.141(-0.022~0.305) | 15.51% | 0.03 |
| PWY-1269 | TG | 0.025(0.001~0.061) * | 0.113(-0.065~0.295) | 0.138(-0.039~0.314) | 15.78% | 0.04 |
| PWY-5913 | CRP | 0.025(0.001~0.058) * | 0.108(-0.060~0.271) | 0.133(-0.026~0.294) | 16.13% | 0.034 |
| PWY-6122 | CRP | 0.028(0.003~0.059) * | 0.104(-0.061~0.264) | 0.132(-0.032~0.297) | 18.00% | 0.026 |
| PWY-6151 | CRP | 0.025(0.003~0.056) * | 0.107(-0.056~0.271) | 0.132(-0.030~0.293) | 15.88% | 0.022 |
| PWY-6277 | CRP | 0.029(0.003~0.066) * | 0.104(-0.069~0.277) | 0.132(-0.034~0.302) | 19.52% | 0.02 |
| PWY-6383 | TG | 0.057(0.020~0.105) *** | 0.090(-0.082~0.273) | 0.147(-0.017~0.322) | 35.72% | 0.00 |
| PWY-6383 | CRP | 0.049(0.012~0.092) ** | 0.083(-0.090~0.249) | 0.132(-0.034~0.299) | 32.76% | 0.008 |
| PWY-6396 | CRP | 0.039(0.011~0.077) ** | 0.095(-0.072~0.270) | 0.135(-0.035~0.311) | 26.94% | 0.004 |
| PWY-6396 | TG | 0.033(0.007~0.069) ** | 0.105(-0.057~0.281) | 0.138(-0.020~0.316) | 21.88% | 0.004 |
| PWY-6470 | TG | 0.040(0.010~0.083) ** | 0.104(-0.051~0.281) | 0.144(-0.017~0.317) | 25.38% | 0.008 |
| PWY-6470 | CRP | 0.035(0.007~0.072) ** | 0.097(-0.063~0.274) | 0.132(-0.030~0.309) | 23.79% | 0.01 |
| PWY-7003 | TG | 0.044(0.011~0.085) ** | 0.098(-0.080~0.278) | 0.142(-0.042~0.316) | 27.90% | 0.002 |
| PWY-7221 | CRP | 0.026(0.002~0.059) * | 0.108(-0.049~0.280) | 0.134(-0.019~0.310) | 16.91% | 0.028 |
| PWY-7237 | TG | 0.041(0.009~0.079) ** | 0.102(-0.072~0.270) | 0.143(-0.034~0.312) | 26.16% | 0.008 |
| PWY1G-0 | TG | 0.042(0.007~0.087) * | 0.100(-0.076~0.280) | 0.142(-0.031~0.329) | 26.07% | 0.018 |
| PWY1G-0 | CRP | 0.040(0.006~0.086) * | 0.101(-0.061~0.271) | 0.141(-0.021~0.307) | 25.74% | 0.014 |

The exposure was smoking. Effect sizes were shown as β (95% confidence intervals). *, 0.01 < p ≤ 0.05, ** 0.001 < p ≤ 0.01, *** p ≤ 0.001. ACME, Average causal mediated effect;ADE, Average direct effect; TE, Total effect; TG, Triglyceride; CRP, C-reactive protein; HDL-C；High-density lipoprotein cholesterol.
